# Association of *IL6* Gene Polymorphisms with COVID-19 Susceptibility and Inflammation in Pregnant Women

**DOI:** 10.3390/diseases14020048

**Published:** 2026-01-30

**Authors:** Imene Ben Dhifallah, Kaouther Ayouni, Ghassen Kharroubi, Zeineb Belaiba, Majdi Ben Ameur, Henda Touzi, Walid Hammemi, Nesrine Abderahmane, Amel Sadraoui, Khaoula Magdoud, Hiba Mkadmi, Samia Kacem, Myriam Cheour, Hajer Chourou, Rim Ben Hmid, Youssef Atef, Khaled Neji, Mohamed Bedis Channoufi, Emna Barkaoui, Dalenda Chelli, Henda Triki, Mariem Gdoura

**Affiliations:** 1 Laboratory of Clinical Virology, Pasteur Institute of Tunis, University of Tunis El Manar, 13 Place Pasteur, BP 74, Tunis 1002, Tunisia; kaouther.ayouni@gmail.com (K.A.); zeineb.belaiba114@gmail.com (Z.B.); majdibenameur9@gmail.com (M.B.A.); touzihenda@yahoo.fr (H.T.); hammemi.walid12@gmail.com (W.H.); nesrine.abde123@gmail.com (N.A.); amelsedraoui1@gmail.com (A.S.); henda.triki@pasteur.tn (H.T.); mariemgdoura@gmail.com (M.G.); 2 Research Laboratory: “Virus, Vector and Host” (LR20IPT02), Pasteur Institute of Tunis, University of Tunis El Manar, 13 Place Pasteur, BP 74, Tunis 1002, Tunisia; 3 Clinical Investigation Center: 2016CICIPT02, Institut Pasteur de Tunis, University of Tunis El Manar, 13, Place Pasteur, Tunis 1002, Tunisia; ghassen.kharroubi@pasteur.tn; 4 Faculty of Sciences of Tunis, University of Tunis El Manar, Campus Universitaire El Manar, Tunis 2092, Tunisia; 5 Laboratory of Medical Epidemiology, Pasteur Institute of Tunis, University of Tunis El Manar, 13 Place Pasteur, BP 74, Tunis 1002, Tunisia; 6 Laboratory of Transmission, Control and Immunobiology of Infections (LR11IPT02), Pasteur Institute of Tunis, University of Tunis El Manar, 13 Place Pasteur, BP 74, Tunis 1002, Tunisia; 7 Department of Gynecology Emergency, Center of Maternity and Neonatology, Boulevard 9 Avril, Tunis 1006, Tunisia; magdoudkhaoula@gmail.com (K.M.); hibamkadmi03@gmail.com (H.M.); rimbenhmid@yahoo.fr (R.B.H.); 8 Department of Neonatal Intensive Care, Center of Maternity and Neonatology, Boulevard 9 Avril, Tunis 1006, Tunisia; samiakacemba@gmail.com (S.K.);; 9 Department of Gynecology, Center of Maternity and Neonatology, Boulevard 9 Avril, Tunis 1006, Tunisia; hajerchourou@gmail.com (H.C.); youssef_atef@yahoo.fr (Y.A.); drkhaledneji21@gmail.com (K.N.); badis.chanoufi@yahoo.fr (M.B.C.); dalendac@yahoo.fr (D.C.); 10 Department of Preventive and Social Medicine, Mother and Child Protection Center, Avenue 2 Mars 1934, Ezzouhour, Tunis 2052, Tunisia; 11 Cellular and Molecular Hematology Laboratory, Pasteur Institute of Tunis, University of Tunis El Manar, 13 Place Pasteur, BP 74, Tunis 1002, Tunisia; 12 Faculty of Medicine of Tunis, University of Tunis El Manar, 15 Rue Djebel Lakhdhar, La Rabta, Tunis 1007, Tunisia

**Keywords:** IL-6, polymorphism, SARS-CoV-2, pregnancy

## Abstract

**Background/Objectives:** Pregnancy is characterized by complex immunological adaptations that may increase susceptibility to infections, including SARS-CoV-2. Interleukin-6 (IL-6), a key pro-inflammatory cytokine, plays a crucial role in the immune response and has been strongly implicated in the pathogenesis of COVID-19. Genetic variations in the *IL6* gene, particularly single-nucleotide polymorphisms (SNPs) in the promoter region, can modulate IL-6 expression and potentially influence individual susceptibility to viral infections. This study aimed to evaluate the relationship between promoter region *IL6* gene polymorphisms and COVID-19 susceptibility, as well as the inflammatory response, in pregnant women. **Methods:** We enrolled in this study 204 pregnant women with evidence of SARS-CoV-2 infection in pregnancy and 134 pregnant women with no evidence of SARS-CoV-2 infection in the past. Genotyping was conducted for the two functional SNPs in the *IL6* promoter region, rs1800796 and rs1800797, via Sanger sequencing, and for associations with COVID-19 susceptibility and IL-6 levels were analyzed. **Results:** No significant association was found between *IL6* polymorphisms and COVID-19, IL-6 levels, age, or immunization status. IL-6 levels > 5 pg/mL were more frequent in SARS-CoV-2-negative pregnant women than in SARS-CoV-2-positive pregnant women (*p* = 0.032). Among vaccinated participants, IL-6 levels were significantly higher in SARS-CoV-2-negative pregnant women (*p* = 0.044), while no difference was observed in the unvaccinated group. **Conclusions:** *IL6* polymorphisms rs1800797 and rs1800796 were not associated with infection susceptibility or IL-6 levels. These results highlight the complex immunological interplay between pregnancy, infection, and genetic background and support the need for further research in larger cohorts.

## 1. Introduction

Since its emergence in December 2019 in Wuhan, China, the coronavirus disease 2019 (COVID-19) pandemic has had a profound global impact, infecting over than 180 million people and causing significant health, economic, and social disruption worldwide [1]. COVID-19 is caused by the severe acute respiratory syndrome Coronavirus 2 (SARS-CoV-2) and presents with a wide spectrum of clinical manifestations, ranging from asymptomatic infection to severe pneumonia and multi-organ failure. Several risk factors for severe disease have been identified, including advanced age, obesity, comorbidities, and pregnancy [2].

Pregnancy involves complex immunological, cardiovascular, and respiratory adaptations that support fetal development but may also increase maternal vulnerability to respiratory infections such as SARS-CoV-2 [3]. Consequently, COVID-19 during pregnancy has been linked to adverse outcomes, including preterm birth, preeclampsia, premature rupture of membranes, admission to intensive care units, and neonatal complications [4]. A prospective study conducted in Tunisia on 11 pregnant women with confirmed SARS-CoV-2 infection reported that most presented with common symptoms such as fever, dry cough, dyspnea, and headache, with two cases developing acute respiratory distress syndrome (ARDS) requiring high-flow oxygen therapy, but no cases of maternal death or vertical transmission were observed [5]. This study investigates the occurrence of SARS-CoV-2 infection among pregnant women.

COVID-19 progression is influenced not only by viral factors but also by the host’s immune response. An inadequate immune response can allow viral dissemination, whereas an exaggerated response may cause tissue damage and multi-organ failure [6,7]. SARS-CoV-2 infects alveolar and gastrointestinal cells by binding to specific receptors, primarily ACE2, but a variety of alternative receptors and co-factors may be involved (such as CD147, Neuropilin-1, CD26, and integrins), activating both innate and adaptive immunity and promoting the release of pro-inflammatory cytokines including interleukin-6 (IL-6) [8,9]. Excessive immune activation can lead to lymphocyte dysfunction, lymphopenia, and worse clinical outcomes [10,11,12]. The severity of COVID-19 is closely associated with immune dysregulation, particularly the excessive release of pro-inflammatory cytokines, a phenomenon known as a cytokine storm [13]. Cells of both the innate and adaptive immune systems, including macrophages, play a key role in the development of cytokine storms observed in various conditions such as Macrophage Activation Syndrome (MAS), cytokine release syndrome associated with Chimeric Antigen Receptor (CAR) T-cell therapy, Severe Acute Respiratory Syndrome (SARS), and Middle East Respiratory Syndrome (MERS) [14,15,16,17,18,19]. Activated macrophages can produce several pro-inflammatory cytokines, such as IL-6, which may trigger the inflammatory cascade, generating a cytokine storm. IL-6 is a 21-kDa glycoprotein encoded by the *IL6* gene, which regulates inflammation and immune responses. Several single-nucleotide polymorphisms (SNPs) in the *IL6* gene, particularly in its promoter region, can affect IL-6 production and contribute to interindividual differences in immune responses [20,21].

Two single-nucleotide polymorphisms (SNPs) located in the promoter region of the *IL6* gene: rs1800796 (−572 G>C) and rs1800797 (−597 G>A) have been among the most frequently investigated *IL6* promoter variants and have shown variable allele frequencies and effects across populations [22]. They were selected based on previous studies reporting their potential functional relevance in modulating *IL6* gene transcription and prior evidence of functional relevance in inflammatory and infectious diseases [23,24]. They have been implicated in the severity of various inflammatory and respiratory diseases, including pneumonia, asthma, and Chronic Obstructive Pulmonary Disease (COPD) [23,24]. These polymorphisms can modulate gene expression and cytokine levels, thereby influencing the immune response to infections like those with influenza virus, hepatitis B virus, and human immunodeficiency virus [25,26]. Their analysis of pregnant women with COVID-19 may therefore provide valuable insight into the genetic factors influencing inflammatory responses during pregnancy.

Despite extensive research on *IL6* polymorphisms in the general population, data remain scarce regarding pregnant women, especially in regions like North Africa, where genetic diversity intersects with health disparities. The North African population, including Tunisians, is characterized by considerable genetic diversity, particularly in genes related to immunity. The Tunisian population exhibits substantial genetic diversity, reflecting its complex demographic history and geographic position at the crossroads of Africa, Europe, and the Middle East [27].

In this context, our study investigates the association between *IL6* promoter polymorphisms (rs1800796 and rs1800797), serum IL-6 levels, and SARS-CoV-2 susceptibility in Tunisian pregnant women, and to our knowledge, this is the first investigation in this field. By exploring how genetic variants may influence immune responses and SARS-CoV-2 susceptibility during pregnancy, this study aims to provide new insights into maternal immunogenetics.

## 2. Materials and Methods

### 2.1. Study Population

Participants consisted of Tunisian pregnant women recruited between 2021 and 2023 at the Centre of Maternity and Neonatology of Tunis (CMNT), which receives pregnant women from the entire Tunis region and the northwestern part of the country. Blood samples from SARS-CoV-2–positive and control pregnant women were collected during prenatal visits and under exceptional circumstances during the COVID-19 pandemic. All analyses were under comparable clinical conditions, ensuring similar timing and physiological status across all participants. All participants shared a similar ethnic and environmental background. SARS-CoV-2 infection was assessed via qRT-PCR on nasopharyngeal swabs, complemented by serological testing for anti-S (spike) and anti-N (nucleocapsid) antibodies using the ROCHE Elecsys^®^ Anti-SARS-CoV-2 assay on the COBAS^®^ e411 analyzer (Roche Diagnostics, Indianapolis, IN, USA). This combined molecular and serological approach enabled the identification of past SARS-CoV-2 infections and differentiation, among vaccinated pregnant women, between natural infection (anti-N positive) and vaccine-induced immunity (anti-S positive, anti-N negative). Participants were classified into two groups: SARS-CoV-2 (+) women with PCR-confirmed SARS-CoV-2 infection during pregnancy and SARS-CoV-2 (–) women with negative qRT-PCR and negative serology (anti-S and anti-N), serving as controls. All SARS-CoV-2–positive pregnant women included in the study experienced mild to moderate COVID-19, with no severe cases observed (mild to moderate cases only, no severe illness observed). Pregnant women with evidence of past infection (RT-PCR negative but seropositive anti-S and/or anti-N in unvaccinated individuals, or anti-N positive in vaccinated women) were excluded. Control participants were pregnant women recruited during the same period, from the same maternity center, with negative qRT-PCR and negative serology (anti-S and anti-N) for SARS-CoV-2 under identical inclusion and exclusion criteria as the infected group, except for SARS-CoV-2 infection status They were clinically healthy, had singleton pregnancies, and were free of chronic inflammatory, autoimmune, or infectious diseases at the time of sampling. All women were in the third trimester of pregnancy, with comparable gestational ages between groups (controls: mean gestational age = 38 weeks; infected: mean gestational age 37 weeks). Control participants were clinically healthy, had singleton pregnancies, and were free of chronic inflammatory, autoimmune, or infectious diseases at the time of sampling. By selecting control participants from the same hospital population, we minimized potential population stratification and environmental variability. This homogeneous recruitment strategy ensures that any observed differences are more likely related to infection status rather than underlying demographic or physiological disparities.

### 2.2. Sample Collection

Peripheral blood samples (5 mL) were collected from each participant in tubes containing EthyleneDiamineTetra-Acetic acid (EDTA). Plasma was separated by centrifugation and stored at −80 °C for IL-6 quantification. The cellular fraction was used for genomic DNA extraction.

### 2.3. IL-6 Quantification

Only serum IL-6 levels were quantified from samples collected at the time of diagnosis in infected women and during prenatal visits in controls. The same analytical procedure was applied to all samples. Serum IL-6 concentrations were determined using the ROCHE Elecsys^®^ IL-6 chemiluminescent immunoassay on the COBAS^®^ e411 analyzer (Roche Diagnostics, Indianapolis, IN, USA), according to the manufacturer’s instructions. All samples were analyzed in duplicate, and the mean value was expressed in pg/mL. A threshold of 5 pg/mL was used to categorize IL-6 protein levels, corresponding to the mean serum concentration observed among all participants in this study.

### 2.4. DNA Extraction and Genotyping

Genomic DNA was extracted from EDTA anticoagulated whole blood using a conventional salting-out procedure or QIAamp DNA blood kit (QIAGEN, Hilden, Germany) following the manufacturer’s instructions. Two functional polymorphisms in the *IL6* gene promoter region, rs1800796 (−634 G>C) and rs1800797 (−597 G>A), were used for genotyping. Specific primers were used to amplify the target regions, forward primer F: 5′-GACTCAGTGGCAATGGGAGAGC-3′ and reverse primer R:5′CGCAAGAAGCAGAACCACTCTTCC-3′ [28]. Amplified DNA was purified using the QIAquick PCR purification Kit (QIAGEN, GmnH, Hilden, Germany) for Sanger sequencing. Sequencing was performed using the Big Dye terminator ready reaction cycle sequencing Kit (Applied Biosystems, Waltham, MA, USA) and both forward and reverse PCR primers on an ABI Prism 3130-Genetic Analyser (Applied Biosystems, Waltham, MA, USA). Genotypes of the study population were then assigned based on nucleotide sequence analysis using Chromas software version 2.6.2.

### 2.5. Statistical Analysis

Statistical analysis was performed using Epistat statistical package. Analysis was conducted to compare age, interleukin level, immunization status and the genotypes and alleles frequencies of rs1800796 and rs1800797 between SARS-CoV-2 positive, and SARS-CoV-2 negative pregnant women, using the standard chi-squared test. Fisher’s exact test was applied when the expected cell number was less than 5. Probability values were considered as statistically significant when *p* < 0.05.

## 3. Results

In total, 338 pregnant women were enrolled in the present work and were divided into two groups based on their SARS-CoV-2 infection status, as determined by serological testing. The group SARS-CoV-2 (+) consisted of 204 women, while the group SARS-CoV-2 (−) included 134 women with no evidence of SARS-CoV-2 infection. The age of our study population ranged from 19 to 45 years (mean: 31, IQR: 27.5–35.5). Table 1 shows the distribution by age, vaccination status, IL-6 levels and *IL6* polymorphisms rs1800796 (−634G>C) and rs1800797 (−597G>A) genotypes in the SARS-CoV-2 (+) and SARS-CoV-2 (−) groups. There was no significant difference according to the age distribution between the two groups (*p* = 0.098). However, the anti-SARS-CoV-2 vaccination status differed significantly between the two groups: 60.4% participants of SARS-CoV-2 (+) group were unvaccinated, while 75.2% those belonging to the SARS-CoV-2 (−) group were vaccinated (*p* < 0.001). The IL-6 level ranged from 1.5 to 152.9 pg/mL (mean: 4.49, IQR: 3.32–8.39). A higher proportion of SARS-CoV-2 negative pregnant women had serum IL-6 levels > 5 pg/mL compared to SARS-CoV-2 positive pregnant women (49.1% vs. 36.5%; *p* = 0.032) (Table 1).

No significant associations were found between IL-6 levels and age (*p* = 0.261) or vaccination status (*p* = 0.187) (Table 2). In the SARS-CoV-2-positive group, IL-6 levels > 5 pg/mL were only more frequent in younger women (≤30 years) (42.6% vs. 30.2%), although the difference did not reach statistical significance (*p* = 0.072). In the same group, vaccination status was also not significantly associated with IL-6 levels (*p* = 0.959).

Table 3 shows that among vaccinated participants, IL-6 levels > 5 pg/mL were significantly more frequent in SARS-CoV-2 negative (52.4%) than in SARS-CoV-2 positive pregnant women (36.7%) (*p* = 0.044). However, in unvaccinated women, no significant difference in IL-6 levels was observed between the two groups (*p* = 0.894) (Table 3).

**Figure 1:**
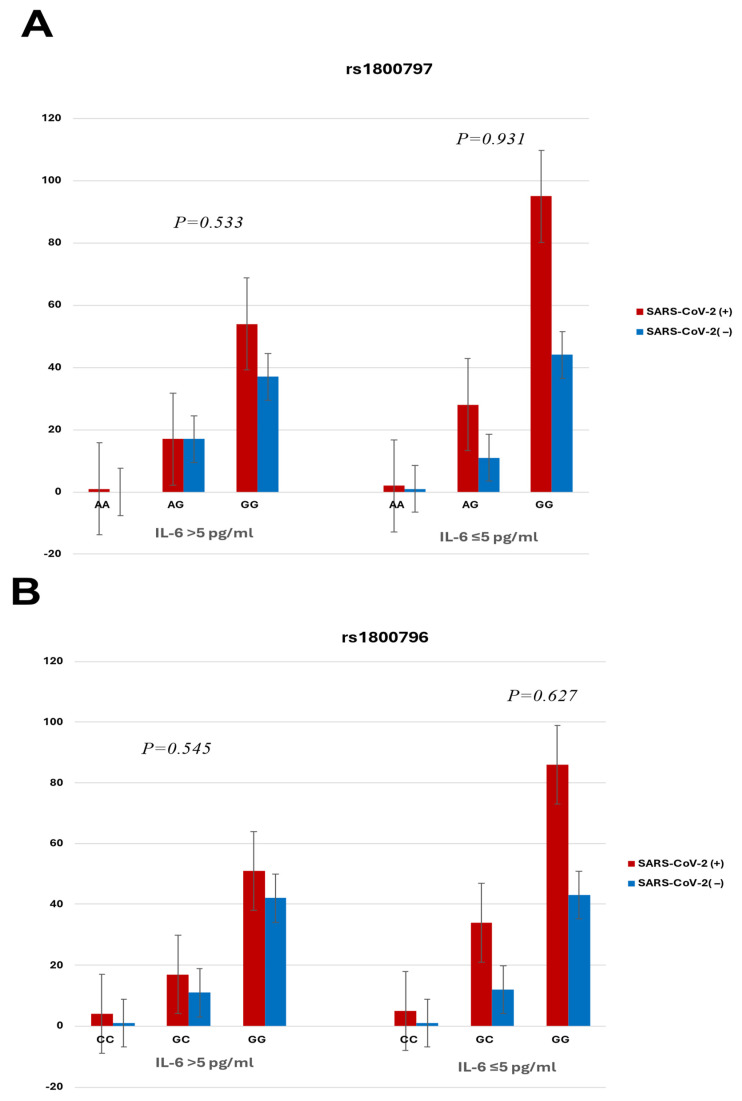
Distribution of rs1800797 (−597G>A) and rs1800796 (−634G>C) genotypes according to IL-6 levels in SARS-CoV-2-positive pregnant women and controls: (**A**) rs1800797 (−597G>A); (**B**) rs1800796 (−634G>C).

The distributions of rs1800797 (−597 G>A) and rs1800796 (−634 G>C) genotypes were in Hardy–Weinberg equilibrium for the two groups (SARS-CoV-2 (+): *p* = 0.926 and 0.140; and SARS-CoV-2 (−): *p* = 0.367 and 0.192 for rs1800797 and rs1800796, respectively). No significant differences were observed in the distribution of *IL6* rs1800797 (−597 G>A) or rs1800796 (−634 G>C) genotypes between infected women and women with no evidence of infection. The AA (rs1800797) and CC (rs1800796) genotypes were rare in both groups (Table 1). Figure 1 and Table 2 analyze the association between *IL6* promoter polymorphisms (rs1800797 −597G>A and rs1800795 −634G>C) and the level of the IL-6 cytokine in SARS-CoV-2 (+) pregnant women and controls. No significant differences in the allele or genotype frequencies were found.

## 4. Discussion

This research should be considered an exploratory pilot study, providing preliminary but valuable evidence on the relationship between *IL6* gene variants and COVID-19 susceptibility in pregnancy. Pregnancy represents a state of altered immunity, characterized by a delicate immunological balance that ensures fetal tolerance while maintaining maternal defense. This unique immune environment may influence the course of infectious diseases, including SARS-CoV-2 infection, which has been associated with adverse pregnancy and neonatal outcomes. The variability in susceptibility to SARS-CoV-2 infection is multifactorial, involving both viral characteristics and host genetic factors, particularly polymorphisms in pro-inflammatory cytokine genes such as *IL6* [29]. IL-6 is a major cytokine in inflammatory response, implicated in cytokine storm syndromes, increased vascular permeability, and multi-organ dysfunction [30,31]. In this study we aimed to evaluate the relationship between promoter region *IL6* gene polymorphisms and COVID-19 susceptibility, as well as the inflammatory response. We also explored how these polymorphisms relate to age and immunization status as potential modifiers of genetic susceptibility. Previous studies have shown that serum IL-6 levels are markedly elevated, up to tenfold, in critically ill COVID-19 patients [31] and that IL-6 levels may serve as a biomarker for disease severity [32,33]. IL-6 is a key cytokine associated with the outcomes of COVID-19, influencing both the duration and severity of the disease [34]. In addition, a systematic review and meta-analysis demonstrated that IL-6 was associated with long COVID-19 [35]. However, Megasari et al. (2024) found that IL-6 levels were not significantly different between mild-to-moderate COVID-19 patients, recovered individuals, and healthy controls [36]. Unexpectedly, we found that the frequency of IL-6 levels exceeding 5 pg/mL was significantly higher in control women compared to those previously infected with SARS-CoV-2 (*p* = 0.032). This finding might be explained by the fact that 75.2% of the control group were vaccinated against COVID-19, whereas only 39.6% of the previously infected women had been vaccinated. Although we did not collect detailed past medical history for participants in the two groups, it is possible that the higher vaccination rate among controls reflects an underlying presence of comorbidities that prompted vaccination. Indeed, previous studies have shown that certain prevalent conditions such as obesity, asthma, and diabetes are associated with increased IL-6 levels [37,38,39].

Polymorphisms in the *IL6* promoter region are known to modulate cytokine gene expression [40,41]. Fishman et al. (1998) first linked the −597G>A variant to enhanced *IL6* transcription [25]. More recently, Liu et al. (2022) confirmed the impact of rs1800796 and rs1800797 on *IL6* transcriptional activity [42]. We found no significant associations between *IL6* polymorphisms (rs1800797 and rs1800796) and SARS-CoV-2 infection status in our study population. Our results show that for rs1800797, genotype frequencies in the general population were GG: 75.0%, AG: 23.9%, and AA: 0.7%, with an A allele frequency of 12.7%. For rs1800796, the genotype frequencies were GG: 76.9%, GC: 20.1%, and CC: 3.0%, corresponding to a C allele frequency of 13.1%. A comparable distribution was observed among previous Tunisian studies and only two studies have documented the genotypic and allelic distributions of these variants in the general population [43,44]. In the study by Mestiri et al. (2020), the reported genotype frequencies for rs1800796 were GG: 64.7%, CG: 28.3%, and CC: 7.0%, corresponding to allele frequencies of G: 79.0% and C: 21.0% [43]. Similarly, Zidi et al. (2017) reported for rs1800797 genotype frequencies of GG: 70.7%, GA: 20.1%, and AA: 9.1%, with allele frequencies of G: 80.8% and A: 19.2% [44]. In our study, the allele frequencies of the *IL6* polymorphisms rs1800797 (A: 12.7%, G: 87.3%) and rs1800796 (C: 13.1%, G: 86.9%) were relatively low. This observation is consistent with previous reports on other *IL6* SNPs in Tunisia, where certain alleles also exhibit low frequencies. Studies have reported G = 96.7% and T = 3.3% for rs2069827, and G = 88.7% and C = 11.3% for rs1800795 and G = 87.8% and C= 12.2% for rs1474348 [44,45]. These data indicate that low-frequency alleles are a common feature among IL-6 polymorphisms across different populations and suggest that the allele distributions observed in our cohort are consistent with the general patterns reported in the literature. Rare genotypes such as AA (rs1800797) and CC (rs1800796) were uncommon in both infected and control groups, reducing statistical power. These findings are in line with several studies. *IL6* SNPs were not associated with COVID-19 severity in Chinese patients [23], nor in the Kurdish population in Iran [28]. In contrast, Amodio et al. (2023) did find a link between *IL6* SNPs and disease severity, though not with susceptibility [46]. Thus, in pregnancy, the role of *IL6* polymorphisms remains inconclusive [46]. As Sayaril et al. (2018) observed increased IL-6 expression in the placenta and blood of women with preterm birth but found a low association between rs1800796 and PTB risk [47]. Similarly, studies by Ramos et al. (2016) [48], Bitner and Kalinka (2010) [49], and Sata et al. (2009) [50] reported no significant associations. In our study, the limited data on neonatal outcomes and delivery mode limited the ability to fully evaluate these clinical associations. For instance, the rs1800797 polymorphism has been previously associated with cervical cancer susceptibility in a Lithuanian population [51], highlighting how the impact of *IL6* variants may differ across diseases and populations. Such inconsistencies across studies may be attributed to differences in sample size, inclusion criteria, disease pathogenesis, ethnicity, and geographical variation.

To further investigate genotype–phenotype correlations, we assessed serum IL-6 levels in relation to genotypes. Stratification based on IL-6 levels (≤5 pg/mL vs. >5 pg/mL) revealed no significant association with any *IL6* polymorphisms. Even among carriers of rare AA or CC genotypes, IL-6 levels did not differ significantly from those with common genotypes. This supports the hypothesis that non-genetic factors such as hormonal, immunologic, or environmental modulators may play a greater role in regulating cytokine expression during pregnancy, even in the context of viral infection [52].

Although combinations such as GA/GC and AA/GC were found exclusively in the infected group, their frequencies were very low. These rare genotypic profiles, although potentially suggestive of a predisposition, did not reach statistical significance (*p* > 0.05), likely due to the small sample size. Taken together, our findings suggest that, in this cohort of Tunisian pregnant women, *IL6* promoter polymorphisms do not significantly influence SARS-CoV-2 infection risk or IL-6 cytokine levels. This study has several limitations that should be acknowledged. It was conducted under exceptional circumstances during the COVID-19 pandemic, when recruitment of pregnant women was particularly challenging due to ethical, logistical, and safety considerations. First, the sample size, although comparable to other genetic studies conducted in pregnant women, may limit the detection of small effect sizes [53,54,55]. A replication in larger cohorts was desirable. Only two functional *IL6* promoter polymorphisms (rs1800796 and rs1800797) were analyzed. Future studies including the three major *IL6* promoter SNPs (rs1800795, rs1800796, and rs1800797) would provide a more comprehensive understanding of the genetic contribution to IL-6 regulation during pregnancy and COVID-19 infection. In addition, other variants, such as rs2069837 or rs2066992, could be considered in future studies to provide a more complete view of *IL6* gene variability. Also, a limitation of our study is the incomplete data on comorbidities, which prevented statistical comparison and adjustment for these potential confounding factors. While this may influence the interpretation of associations between *IL6* polymorphisms and cytokine levels, the study population was relatively homogeneous between patients and controls, and the findings remain biologically plausible. Finally, IL-6 serum levels were measured at a single time point, which may not fully capture the dynamic changes in inflammatory response during pregnancy and infection. Taken together, these limitations do not undermine the validity of the results but rather highlight the challenges and value of conducting genetic studies in pregnant populations during a global pandemic.

## 5. Conclusions

This work provides novel insight into *IL6* genetic variability and immune modulation during pregnancy in an underrepresented North African population. While SARS-CoV-2 is known to drive IL-6 expression, our results suggest that in the context of pregnancy, immune activation can be more complex and not solely determined by active viral infection. Our findings reinforce the complexity of immune regulation during pregnancy and suggest that IL-6 expression is influenced more by dynamic environmental and epigenetic factors than by promoter polymorphisms alone. Further longitudinal studies are needed to distinguish between infection-related inflammation and pregnancy-related immune modulation.

## Figures and Tables

**Table 1:** Demographic Characteristics and *IL6* SNP rs1800797 −597 G>A and rs1800796 −634 G>C genotype frequencies in SARS-CoV-2-positive and -negative pregnant women.

	SARS-CoV-2 (+) n (%)	SARS-CoV-2 (−) n (%)	*p*-Value
**Age class**			
>30 years	99 (48.5)	68 (58.1)	0.098
≤30 years	105 (51.5)	49 (41.9)	
**Vaccination Status**			
Vaccinated	80 (39.6)	97 (75.2)	**<0.001**
Unvaccinated	122 (60.4)	32 (24.8)	
**IL-6 class**			
>5 pg/ml	72 (36.5)	54 (49.1)	**0.032**
≤5 pg/ml	125 (63.5)	56 (50.9)	
**rs1800797 (−597 G>A)**			
AA	4 (2.0)	1 (0.7)	0.802
AG	48 (23.5)	32 (23.9)	
GG	152 (74.5)	101 (75.0)	
A	56 (13.7)	34 (12.7)	0.697
G	352 (86.3)	234 (87.3)	
**rs1800796 (−634 G>C)**			
CC	9 (4.4)	4 (3.0)	0.382
GC	52 (25.5)	27 (20.1)	
GG	143 (70.1)	103 (76.9)	
C	70 (17.2)	35 (13.1)	0.150
G	338 (82.8)	233 (86.9)	

Bold value: significant *p* value (*p*-value < 0.05)

**Table 2:** Distribution of serum IL-6 levels among pregnant women according to demographic data and genotype frequencies of *IL6* SNP rs1800797 (−597 G>A) and rs1800796 (−634 G>C).

	SARS-CoV-2 (+)			SARS-CoV-2(−)			Total		
	IL-6 > 5 pg/mL n (%)	IL-6 ≤ 5 pg/mL n (%)	*p*-Value	IL-6 > 5 pg/mL n (%)	IL-6 ≤ 5 pg/mL n (%)	*p*-Value	IL-6 > 5 pg/mL n (%)	IL-6 ≤ 5 pg/mL n (%)	*p*-Value
**Age class**									
>30 years	29 (30.2)	67 (69.8)	0.072	31 (50.0)	31 (50.0)	0.828	60 (38.0)	98 (62.0)	0.261
≤30 years	43 (42.6)	58 (57.4)		23 (47.9)	25 (52.1)		66 (44.3)	83 (55.7)	
**Vaccination Status**									
Vaccinated	29 (36.7)	50 (63.3)	0.959	44 (52.4)	40 (47.6)	0.215	73 (44.8)	90 (55.2)	0.187
Unvaccinated	43 (37.1)	73 (62.9)		10 (38.5)	16 (61.5)		53 (37.8)	89 (62.7)	
**rs1800797 (−597 G>A)**									
AA	1 (33.3)	2 (66.7)	0.936	0 (0)	1 (100)	0.191	1 (25.0)	3 (75.0)	0.521
AG	17 (37.8)	28 (62.2)		17 (60.7)	11 (39.3)		34 (46.6)	39 (53.4)	
GG	54 (36.2)	95 (63.8)		37 (45.7)	44 (54.3)		91 (39.6)	139 (60.4)	
A G	19 (13.2) 125 (86.8)	32 (12.8) 218 (87.2)	0.910	17 (15.7) 91 (84.3)	13 (11.6) 99 (88.4)	0.372	36 (14.3) 216 (85.7)	45 (12.4) 317 (87.6)	0.504
**rs1800796 (−634 G>C)**									
CC	4 (44.4)	5 (55.6)	0.800	1 (50.0)	1 (50.0)	1.000	5 (45.5)	6 (54.5)	0.807
GC	17 (33.3)	34 (66.7)		11 (47.8)	12 (52.2)		28 (37.8)	46 (62.2)	
GG	51 (37.2)	86 (62.8)		42 (49.4)	43 (50.6)		93 (41.9)	129 (58.1)	
C	25 (17.4)	44 (17.6)	0.952	13 (12.0)	14 (12.5)	0.917	38 (15.1)	58 (16.0)	0.752
G	119 (82.6)	206 (82.4)		95 (88.0)	98 (87.5)		214 (84.9)	304 (84.0)	

*p*-value: significant *p*-value (*p*-value < 0.05).

**Table 3:** Distribution of serum IL-6 levels among vaccinated and unvaccinated pregnant women.

	Vaccinated			Unvaccinated		
	IL-6 > 5 pg/mL n (%)	IL-6 ≤ 5 pg/mL n (%)	*p*-Value	IL-6 > 5 pg/mL n (%)	IL-6 ≤ 5 pg/mL n (%)	*p*-Value
**SARS-CoV-2 (+)**	29 (36.7)	50 (63.3)	**0.044**	43 (37.1)	73 (62.9)	0.894
**SARS-CoV-2 (−)**	44 (52.4)	40 (47.6)		10 (38.5)	16 (61.5)	

Bold value: significant *p* value (*p*-value < 0.05)

## Data Availability

The data presented in this study are available upon request from the corresponding author.

## Abbreviations

The following abbreviations are used in this manuscript:

COVID-19	Coronavirus Disease 2019
SARS-CoV-2	Severe Acute Respiratory Syndrome Coronavirus 2
ARDS	Acute Respiratory Distress Syndrome
IL-6	Interleukin 6
SNP	Single-Nucleotide Polymorphism
COP	Chronic Obstructive Pulmonary Disease
MAS	Macrophage Activation Syndrome
CAR T-cell therapy	Chimeric Antigen Receptor T-cell therapy
SARS	Severe Acute Respiratory Syndrome
MERS	Middle East Respiratory Syndrome
HCC	Hepatocellular Carcinoma
HBV	Hepatitis B Virus
HCV	Hepatitis C Virus
PTB	Preterm Birth
EDTA	EthyleneDiamineTetraacetic Acid
CMNT	Centre of Maternity and Neonatology of Tunis
